# Supplementary material for: Confirmation of the Applicability of Skeletochronology and Estimating the Age Structure of Kaloula borealis (Microhylidae: Anura) at Lake Sihwa, South Korea
Source: Biology (Basel). 2022 Jun 10;11(6):898. doi: 10.3390/biology11060898 (PMC9220234; doi:10.3390/biology11060898)
Supplement: Supplementary file 1 [file biology-11-00898-s001.zip › biology-1757600-sup/biology-1757600-supplementary.pdf]

Table S2: Detailed method of Skeletochronology.

S2.1 Pre-processing:

Remove fixing agent – softening – remove softening solution – dehydration – clearing – penetration

Number	Material	Time	Reason	Contents and Precautions
1	Running water	24h	Remove fixing agent	* The best method is to purchase and use a plastic container for tissue staining<br><br>* Separately wrapped with gauze<br>→ About 2 layers is suitable<br><br>* Mark the number with a pencil (graphite)<br>→ Insoluble in organic solvents
2	5% nitric acid solution	2h	Bone softening:<br>The process of softening bones within the tissue	
3	Running water	24h	Remove softening solution	
4	70% Ethanol	30m	Dehydration	* Beware of changes in ethanol concentration: it needs to be replaced regularly
5	80% Ethanol	30m		
6	90% Ethanol	30m		
7	100% Ethanol	30m		
8	100% Ethanol	30m		
9	Histoclear	30m	Clearing:	* Use of undiluted solution
10	Histoclear	30m		

11	Histoclear	30m	The process of substituting a substance that can be fused with a penetrant (paraffin)	
		2d 6h		

S2.2 Embedding process:

The process of preparing the tissue that has been penetrated with a shape suitable for thin cutting using a microtome.

Number	Material	Time	Reason	Contents and Precautions
1	Liquid paraffin in a drying oven set at 60°C to 70°C	24h	Impregnation:<br>Process of paraffin penetration into toe tissue	* Temperature control of the drying oven is important
2	Preparation of a plastic mold (base mold for histology) containing liquid paraffin		Fixing toe tissue	* Appropriate depth of base mold is 5~6mm<br>* Proceed on the pad above the dryer
3	Before the paraffin hardens, the toe tissue is erected vertically using a dissecting needle		Easy microtomy	* If you make a mistake, put it back in the dryer to melt the paraffin and try again<br>* Be careful not to get air around the toe tissue.
4	When the paraffin hardens, it is separated from the plastic mold			
5	Remove excess paraffin in trapezoidal shape, leaving it only around the toe tissue			* The trapezoid should be continuously attached to the blade when cutting

S2.3 Microtomy process:

The process of thinly cutting the paraffin pieces after the embedding process has been completed.

Number	Material	Time	Reason	Contents and Precautions
1	Cut to a thickness of 10-15 ㎛ using a microtome		For observation by staining	* The thinner the better
2	The cut toe cross-section is placed on a slide glass using distilled water			* Use of distilled water<br>* Prepare slide glass
3	Put it in a slide warmer set at 35~40℃	24h	To make sure that the paraffin of the toe cross-section is firmly fixed to the slide glass	* Caution<br>1. If separated:<br>(If paraffin and toe cross-section are separated)<br>There is a problem with the thickness and angle of the blade when cutting<br>2. If torn:<br>There is a problem with the position (bluntness) of the blade<br>3. If there is a hole:<br>(Tissue and bone are separated)<br>There is a problem in the dehydration (pre-treatment) process<br><br>-> If the above problems occur, start

				over from the embedding process.

S2.4 Staining process:

H&E staining method (hematoxylin and eosin staining method) to confirm the age line.

Paraffin removal process → hydrous process → staining process

Number	Material	Time	Reason	Contents and Precautions
1	Histoclear	3m	Paraffin removal process	* Use of undiluted solution
2	Histoclear	3m		* Use of undiluted solution
3	Histoclear	3m		* Use of undiluted solution
4	100% Ethanol	2m	Hydrous process	
5	100% Ethanol	2m		
6	95% Ethanol	1m		
7	95% Ethanol	1m		
8	70% Ethanol	2m		
9	70% Ethanol	2m		
10	Distilled water	5m		
11	100% Ethanol	2m	Staining process	
12	95% Ethanol	2m		
13	95% Ethanol	2m		
14	Scott's tap water substitute	Rinse		
15	Harris's Hematoxylin	30m		* Replace when the stain becomes black
16	Scott's tap water	10th dipping		
17	Scott's tap water	10th dipping		
18	Differentiator	15th dipping		
19	Distilled water	Rinse		* Wash
20	0.05% Lithium Carbonate	30sec		
21	Scott's tap water	10th dipping		* Dipping
22	Scott's tap water	10th dipping		

23	Eosin	15th dipping		* Replace when the stain becomes black
24	95% Ethanol	1m	Clearing	
25	95% Ethanol	1m		
26	100% Ethanol	1m		
27	100% Ethanol	1m		
28	100% Ethanol	1m		
29	Histoclear	3m		* Use of undiluted solution
30	Histoclear	3m		* Use of undiluted solution
31	Histoclear	3m		* Use of undiluted solution
		Almost 1h30m		

S2.5 Mounting and Observation:

Completion of permanent preparation using mounting agent (permount) and age estimate using optical microscope.

Number	Material	Contents and Precautions
1	Completion of permanent preparation using a fixing mounting agent (permount) and cover glass	* Put the mounting agent drop by drop between the samples, and then cover the cover glass
2	Observation with an optical microscope (×400 magnification)	* LAG (purple) / tissue (red)<br>* Check whether Eosin is altered or not
3	Take pictures using an image system and a digital camera	

S2.6 Method of making staining solution

**Serial Number**	**Staining solution**	**Procedure**
**1**	**Harris's Hematoxylin:**<br>**Liquid products can be purchased and used if they are available the offline**	① Completely dissolve 2.5 g of Hematoxylin crystal (C16H14O6) in 100% ethanol (C2H5OH).
		② Dissolve Ammonium potassium sulfate [Alk(SO4)2·12H2O] in 500ml of distilled water and heat.
		③ While heating (before boiling), slowly pour (1) into (2) and mix the two solutions well.
		④ Boil the solution while adding 1.25 g of Mercury (II) oxide (MgO) to the mixed solution.
		⑤ Boil the solution to which all reagents have been added until it turns black purple, and immediately after boiling, put it in a bowl of cold water until it cools

		down.
		⑥ When the solution is cold, add 4 ml of acetic acid (CH_3COOH) per 100 ml to increase the color accuracy.
**2**	**Eosin**	Add 4g of Eosin Y ($C_{20}H_6Br_4Na_2O_5$) to 400ml of distilled water and mix well.
**3**	**Differentiator**	Add 4 ml of hydrochloric acid (HCl) to 400 ml of 70% ethanol (C_2H_5OH).
**4**	**Scotti's tap water substitute**	Add 40g of sodium bicarbonate ($NaHCO_3$) and 7g of magnesium sulfate ($MgSO_4$) to 1L of distilled water and mix well.
